# Proton Pump Inhibitors and Cancer Risk: A Comprehensive Review of Epidemiological and Mechanistic Evidence

**DOI:** 10.3390/jcm13071970

**Published:** 2024-03-28

**Authors:** Ibrahim O. Sawaid, Abraham O. Samson

**Affiliations:** Azrieli Faculty of Medicine, Bar Ilan University, Safed 1311502, Israel; ibrahim.sawaid@biu.ac.il

**Keywords:** CCKB2R—cholecystokinin B2 receptor, ECL cells—enterochromaffin-like cells, GERD—gastroesophageal reflux disease, GI—gastrointestinal, H2R—histamine H2 receptor, H2RB—H2R blocker, NET—neuroendocrine tumor, *Helicobacter pylori*—*H. pylori*, PPI—proton pump inhibitor

## Abstract

**Background:** Proton pump inhibitors (PPIs) are commonly prescribed long-acting drugs used to treat acid reflux, gastroesophageal reflux disease (GERD), and peptic ulcers. Recently, concerns have been raised about their safety, particularly due to the association between long-term PPI use and cancer development. Multiple comprehensive studies have consistently suggested a noteworthy link between prolonged PPI usage and an increased risk of developing gastric, esophageal, colorectal, and pancreatic cancers, yet the precise underlying mechanism remains elusive. **Methods:** First, we review the extensive body of research that investigates the intricate relationship between cancer and PPIs. Then, we predict PPI toxicity using the prodrug structures with the ProTox-II webserver. Finally, we predict the relative risk of cancer for each PPI, using PubMed citation counts of each drug and keywords related to cancer. **Results:** Our review indicates that prolonged PPI use (exceeding three months) is significantly associated with an elevated risk of cancer, while shorter-term usage (less than three months) appears to pose a comparatively lower risk. Our review encompasses various proposed mechanisms, such as pH and microbiome alterations, vitamin and mineral malabsorption, hypergastrinemia, and enterochromaffin-like cell proliferation, while ProTox-II also suggests aryl hydrocarbon receptor binding. Potentially, the PubMed citations count suggests that the PPIs omeprazole and lansoprazole are more associated with cancer than pantoprazole and esomeprazole. In comparison, the H2R blocker, famotidine, is potentially less associated with cancer than PPIs, and may serve as a safer alternative treatment for periods beyond 3 months. **Conclusions:** Despite the well-established cancer risk associated with PPIs, it is notable that these medications continue to be widely prescribed for periods longer than 3 months. Thus, it is of paramount importance for clinicians and patients to thoughtfully evaluate the potential risks and benefits of long-term PPI usage and explore alternative treatments before making informed decisions regarding their medical management.

## 1. Introduction

Proton pump inhibitors (PPIs) are a class of drugs that are used in the treatment of acid reflux, gastroesophageal reflux disease (GERD), and peptic ulcers [1]. Proton pump inhibitors (PPI) reduce stomach acid production through the irreversible and long-acting inhibition of the stomach’s H^+^/K^+^ ATPase proton pumps, which can relieve symptoms such as heartburn, indigestion, and nausea [2]. PPIs are widely prescribed, with millions of people taking them on a daily basis [3].

PPIs were first introduced in the late 1980s as a new class of drugs for the treatment of acid reflux and related conditions. The very first PPI, omeprazole, was developed by scientists of the Swedish pharmaceutical company Astra AB (later AstraZeneca) and was approved for use in that country in 1988. Since the introduction of omeprazole, several other PPIs have been developed, including lansoprazole, dexlansoprazole, pantoprazole, rabeprazole, and esomeprazole. These drugs are currently some of the most commonly prescribed drugs in the world [4].

Before the advent of PPIs, the main treatments for acid reflux and related gastrointestinal (GI) conditions were antacids and histamine 2 receptor (H2R) blockers. Antacids, like Tums, Gaviscon, and Reni (containing salts of CO_3_^2−^), act by neutralizing the hydrochloric acid in the stomach. H2R blockers, like cimetidine, famotidine, nizatidine, and ranitidine, act by blocking the action of histamine, which stimulates acid production in the stomach. While these treatments are effective in reducing acid reflux, they have several limitations, such as a short duration of action. Recently, ranitidine was withdrawn from the market due to product contamination with the carcinogenic N-nitrosodimethylamine [5]. While famotidine and cimetidine remain on the market, cimetidine interacts negatively with multiple other drugs, and famotidine is considered the only relatively safe drug [6].

PPIs revolutionized the pharmacological treatment of acid reflux and related conditions by offering an effective and long-lasting solution. PPIs are effective at decreasing acid production in the stomach, and some patients find them convenient alternatives to antacids and H2R blockers [7]. PPIs are commonly thought to exhibit fewer side effects than antacids and H2R blockers [8]. Despite their many benefits, PPIs are not without risks. Long-term use of PPIs has been associated with an increased risk of mineral (i.e., Ca, Mg, Fe, and Zn) and vitamin (i.e., B12, C, and D) deficiencies, anemia, bone fractures, liver and kidney disease, *Clostridium difficile* infection, non-typhoid *Salmonella* infection, *Campylobacter* infection, *Enterococci* overgrowth, community-acquired pneumonia, dementia, hypergastrinemia, gastric hyperplasia and metaplasia, and cancer [9]. However, for some, the benefits of PPIs outweigh their potential risks, and they persist as an important treatment option for acid reflux and related conditions [10].

In recent years, concerns have accumulated about PPIs, particularly regarding the long-term risks associated with PPI use, including an increased risk of certain types of cancer [11,12,13,14,15,16], such as gastric, esophageal, colorectal, and pancreatic cancers. Concerns have also been raised about a potential link between using PPIs and an increased risk of neuroendocrine tumors (NETs) [17]. Notably, the frequency of the latter in the gastrointestinal tract, and in particular gastric NETs, has shown a significant rise over the past three decades, in parallel with the worldwide advent of PPIs.

The link between PPIs and cancer has raised concerns among healthcare professionals and patients alike [18]. Although PPIs are considered safe for short-term use (<3 months) for treating acid reflux and related conditions, their long-term use may increase the risk of adverse effects. Notably, PPIs are also associated with many benefits, such as improved life quality and reduced risk of complications associated with acid reflux and GERD [19].

Here, we review the literature for papers reporting PPI carcinogenicity and predict the relative risk for cancer due to use of omeprazole, lansoprazole, dexlansoprazole, pantoprazole, rabeprazole, and esomeprazole. Our hypothesis is that prolonged proton pump inhibitor (PPI) usage is significantly correlated with heightened cancer risk, particularly in the context of gastric, esophageal, colorectal, and pancreatic cancers. While also suggesting safer alternatives, such as famotidine, we emphasize the importance of thoughtful evaluation by clinicians and patients when considering long-term PPI use.

## 2. Methods

Literature review. To find papers related to PPIs and cancer, PubMed was consulted using the search words: (“proton pump inhibitor”) AND (“cancer” OR “neoplasm” OR “tumor”). Of these 1000+ papers, a wide selection was reviewed.

PubMed count. To predict the relative risk of cancer of each PPI, we counted the number of PubMed citations of each of the drugs and keywords related to cancer: (“cancer” OR “neoplasm” OR “tumor”). In addition, we counted the number of PubMed citations for each PPI alone.

Toxicity prediction. To predict the toxicity of each PPI, the structure of each PPI was uploaded to the toxicity prediction server (https://comptox.charite.de/protox3 accessed on 1 March 2023). The toxicity prediction server uses chemical formulas to forecast organ toxicity and to estimate receptor binding.

## 3. Results

PPI association with neuroendocrine tumors of the gastrointestinal tract. The gastrointestinal (GI) tract comprises a large number of neuroendocrine cells. Neuroendocrine cells monitor the release of digestive juices, control peristalsis, and regulate the growth of other cell types of the digestive system. Neuroendocrine tumors (NETs) are rare and account for ~1% of stomach tumors, but around 5% of NETs start in the stomach. NETs are more common in females and in people over 60 years old. NETs and carcinoid tumors (i.e., a slow-growing type of NET) have attracted considerable interest within the medical community. Their occurrence has significantly risen over the past few decades. Remarkably, this increase roughly corresponds with the global surge in the usage of PPIs [17]. A study by Dasari and coworkers investigated the association between long-term use of PPIs and the risk of carcinoids. Their study revealed that from 1973, just before the advent of acid-suppressive medications, and until 2012, a total of 64,971 NET cases were documented in North America. During this time, age-adjusted incidence rates exhibited a striking increase—from 1.09 to 6.98 per 100,000 individuals. This increase was observed consistently across various tumor sites, stages, and grades. Particularly striking was the surge in patients aged 65 and older, with an increase to 25.3 cases per 100,000. Furthermore, rectal, intestinal, broncho-pulmonary, pancreatic, and other carcinoids also displayed significant upticks in their occurrence [20]. Notably, this trend of increased incidence in carcinoid tumors of the gastrointestinal tract was also mirrored in the UK over a corresponding period [21]. The surge in gastric carcinoids is even more remarkable when juxtaposed against the distribution of carcinoid tumors diagnosed over the preceding half-century, before the rapid increase [22]. Recently, a study by Prado and coworkers described a case of a NET in a 29-year-old patient with long-term use of PPIs [23], and there are now more than a dozen case reports of gastric carcinoid tumors in patients using a PPI [24,25,26,27,28,29,30,31]. Altogether, the authors express concern about the potential association between hypergastrinemia induced by PPIs and the development of NETs. 

PPI association with cancer. Several original studies have investigated the association between PPI use and cancer, and we review them in the following paragraphs. Some studies have found a positive association between PPI use and an increased risk of cancer, while others have found little or no association (Table 1). It is important to note that the studies investigating the link between PPIs and cancer are observational and cannot prove causation.

A study by Sørensen and coworkers investigated the association between long-term PPI use and the risk of colorectal cancer. The study included 5589 patients diagnosed with colorectal cancer in 1989–2005 and 55,890 age- and sex-matched controls from the general population. The study showed that long-term PPI use was not associated with an increased risk of colorectal cancer among those who had used PPIs for more than 7 years compared to those who had never used them [32].

A study by Al-Aly and coworkers investigated the association between long-term use of PPIs and the risk of death in a large cohort. The study included data from over 6 million patients who received medical care from the veterans affairs healthcare system. The study showed that long-term PPI use was associated with a 25% increased risk of death among those who had used PPIs for more than a year compared to those who had never used them. The study also found a dose-dependent risk of death among PPI users. Notably, the study found that the increased cause of death was not limited to gastrointestinal diseases alone, but also to cardiovascular, renal, and infectious diseases [33].

A study by Leung and coworkers investigated the association between long-term PPI use and the risk of gastric cancer. The study was conducted with 63,000 patients treated for *Helicobacter pylori* (*H. pylori*) infection in Hong Kong, with an average follow-up time of 7.6 years. The study revealed that long-term use of PPIs was associated with a two-fold increased risk of gastric cancer among patients who had used PPIs for more than 3 years compared to those who had never used them. Their study also found a dose-dependent risk of gastric cancer among PPI users [13].

A study by Yang and coworkers investigated the association between PPI use and the risk of female cancers, such as breast, ovarian, cervical, and endometrial cancer. The study was conducted using data of 23 million patients in the database of Taiwan’s Health and Welfare Data Science Center for the years 2000–2016, and was cross-referenced with cancer incidence data of the Taiwan Cancer Registry for the years 1979–2016. Patients without any cancer diagnosis during the 17 years of the study served as controls. The study found that PPIs were associated with an insignificant increase in the risk of breast cancer in the population above 65 years of age. Conversely, the study also found that PPIs were associated with a minor but significant reduced risk of breast, ovarian, and cervical cancer in the population aged 20–65 years [16]. As a limitation to this study, long-term PPI use was defined as 60 days and higher, unlike other studies listed here that define long-term PPI use as 3 months, or even one year. This limitation could have resulted in the discrepancy in results.

A study by Misslewitz and coworkers investigated the association between PPI use and the risk of gastric cancer. The study screened 2396 records and included five retrospective cohort and eight case-control studies comprising 1,662,881 individuals. The study found that long-term use of PPIs was associated with a 1.5-fold increase in the risk of gastric cancer among patients who had used PPIs for more than a year compared to those who had never used them. However, this association increased by 2.4-fold in patients who had used PPIs for more than three years. Notably, this study accounted for age, sex, smoking, and *H. pylori* infection [34].

A study by Stehouwer and coworkers investigated the association between PPI use and the risks of fundic gland polyps and gastric cancer. The study analyzed data from 12 studies, comprising more than 87,324 patients. The study showed that PPIs were associated with an increased risk of fundic gland polyps (2.98-fold) and gastric cancer (1.41-fold). In addition, the risk was higher with long-term PPI use (>1 year) compared to short-term use. The study also found that the risk of fundic gland polyps was highest among patients with high-dose PPIs, a history of *H. pylori* infection, or a family history of gastric cancer [14].

A study by Jung and coworkers investigated the association between PPI use and the risk of pancreatic cancer. The study screened 10 observational studies (7 cohort studies and 3 case-control studies) including 1,259,506 patients. The study showed that PPI use was not significantly associated with the risk of pancreatic cancer (1.08-fold), based on the duration of use, dose, or type of PPI. As a potential limitation, the study did not define long-term PPI use in terms of time, which could scramble the data [35].

PPIs in cancer therapy. PPIs could also have important antitumor effects, as has been reviewed elsewhere [36]. A variety of action mechanisms have been proposed and include the following: immunopotentiation [37], sensitization to chemoradiotherapy [38], the promotion of apoptosis and suppression of migration [39], detoxification inhibition [40], and the suppression of exosomes carrying microRNA [41], among others. 

Conversely, PPI concomitant treatment also leads to significantly worse outcomes in advanced cancer patients [42], increases mortality risk [43], increases risk of disease progression [44] {, and reduces overall survival [45].

Finally, PPI concomitant therapy also does not appear to be significantly associated with overall survival [46] and is uncorrelated with progression-free survival [47].

Considering the inconsistent findings, the antitumor effect of PPIs is more likely to be derived from a combination of factors such as cancer type and stage, as well as concomitant drug therapy [48]. Thus, until a clear pattern emerges, caution should be applied when administering PPIs and systemic antitumor therapy.

Proposed mechanism of cancer. Several studies have suggested a link between long-term PPI use and an increased risk of cancers, but the underlying mechanism remains elusive. 

As a potential mechanism by which PPIs contribute to cancer development is their effect on the gut microbiome [49]. The gut microbiome is a diverse ecosystem of bacteria living in the intestines and is important in maintaining human health [50]. PPIs increase the gut pH and may potentially alter the gut microbiome, reduce the diversity and abundance of beneficial bacteria, and lead to gastrointestinal inflammation. In turn, this could promote the overgrowth of harmful bacteria that produce carcinogens and increase the risk of cancer, such as colorectal cancer [51].

Another possible mechanism by which PPIs contribute to cancer development is their effect on the hormone gastrin. As PPIs increase the pH of the stomach, they can also lead to hypergastrinemia, a pathological condition characterized by the over-secretion of gastrin [52]. Gastrin stimulates acid production [53], and high levels of it have been associated with the proliferation and hyperfunction of enterochromaffin-like (ECL) cells [15] in a concentration-dependent manner, and it is closely associated with long-term exposure [54,55,56] and an increased risk of cancer, such as gastric cancer [57]. The effect is mediated by gastrin/Cholecystokinin B2 receptor (CCKB2R) on the ECL cell membrane, which in turn leads to hyperplasia, dysplasia, and finally neoplasia [58]. Conversely, PPIs could also result in the under-secretion of gastrin and prevent the growth of gastric cells that offer protection against gastric cancer [59].

Another proposed mechanism by which PPIs contribute to cancer development is the proliferation of enterochromaffin-like cells in the stomach, which have been associated with cancer initiation. These specialized cells have been observed to undergo abnormal growth and cellular changes under the influence of prolonged PPI use. Furthermore, the cumulative effects of such alterations in the gastric environment may significantly contribute to the progression of cancer in susceptible individuals [17].

Here, we hypothesize that PPI toxicity is mediated through specific binding to macromolecules involved in the initiation, promotion, and invasion of cancer cells. For example, the toxicity prediction server (https://comptox.charite.de/protox3 accessed on 1 March 2023) proposes that PPIs are all carcinogenic, immunotoxic, and hepatotoxic. In addition, all PPIs are expected to bind to the aryl hydrocarbon receptor (AhR). AhR is an important cytosolic, ligand-dependent transcription factor, and evidence suggests its role in the initiation, promotion, progression, invasion, and metastasis of cancer cells [60]. The toxicity prediction server also suggests that PPIs toxicity could be mediated by binding to the Androgen Receptor, Androgen Receptor Ligand Binding Domain, Aromatase, Estrogen Receptor Alpha, Estrogen Receptor Ligand Binding Domain, Peroxisome Proliferator Activated Receptor Gamma, Nuclear factor like 2/antioxidant responsive element, Heat shock factor response element, Mitochondrial Membrane Potential, Phosphoprotein p53, etc. In addition, predictions were performed using the PPI prodrug structure form, and we hypothesized that PPIs’ active form could bind covalently and non-specifically to unpaired cysteines of proteins and modulate their normal activity [61]. As a potential pitfall, these hypotheses require experimental confirmation, and it is likely that the mechanism of action is a combination of all earlier propositions.

Predicted relative risk of cancer. To predict the relative risk of cancer of each PPI, we consulted PubMed for each of the drugs and keywords related to cancer. Table 2 presents the number of PubMed citations for each PPI drug alone and in combination with the keywords ‘cancer’, ‘neoplasm’, or ‘tumor’. The normalized association was calculated using the following equation:

$$Normalized\;association=\frac{{PubMed\;citations}_{PPI\;and\;(cancer\;OR\;neoplasm\;OR\;tumor)}}{{PubMed\;citations}_{PPI}}$$

The equation accounts for the potential over-representation of highly cited PPI drugs by dividing it by the number of PubMed citations of the PPI alone, thus normalizing the number of PubMed citations of PPIs with cancer keywords. Interestingly, omeprazole (0.12) and lansoprazole (0.12) are potentially more associated with cancer than pantoprazole (0.10) and esomeprazole (0.10). This finding indicates that esomeprazole and pantoprazole are potentially safer than omeprazole and lansoprazole in long-term treatment with PPIs (>3 months). The findings do not distinguish between the cancer association of omeprazole and lansoprazole. Likewise, the findings do not distinguish between pantoprazole and esomeprazole. Importantly, the association for rabeprazole and dexlansoprazole is statistically insignificant due to the small number of citations with keywords (i.e., <200). In comparison, famotidine (0.08), an H2R blocker, is less associated with cancer compared to all of the PPIs and may potentially serve as a safer alternative treatment for periods extending beyond 3 months. In summary, the predicted relative cancer risk of each PPI is thus ranked as following: [Omeprazole and Lansoprazole] > [Pantoprazole and Esomeprazole] > Famotidine. 

As a potential caveat, the relative risk of cancer association cannot be accurately predicted on the basis of citations. Moreover, the prediction does not differentiate between positive and negative associations found in these citations, or the strength and quality of evidence. Nevertheless, the signal-to-noise ratio increases with the number of citations, and large numbers can provide a qualitative prediction of trends. As such, it is crucial to emphasize that these findings are preliminary and await experimental verification. 

While these results could attest to the lower risk of developing cancer associated with pantoprazole and esomeprazole, clinical decisions should always be based on evidence-based medicine. As PPI research advances, so too will our understanding of their potential implications for cancer risk. Furthermore, long-term clinical studies, comparing the benefits and disadvantages to H2R blockers, such as famotidine, are imperative to provide a clearer picture of these associations, ultimately guiding more informed treatment decisions for patients suffering from chronic acid reflux.

## 4. Discussion

Our review indicates that prolonged use of proton pump inhibitors (PPIs) for more than three months is linked to a higher risk of cancer, whereas shorter-term usage (less than three months) appears to carry a comparatively lower risk. We have examined various proposed mechanisms underlying the association between PPIs and cancer risk, including pH and microbiome alterations, vitamin and mineral malabsorption, hypergastrinemia, and enterochromaffin-like cell proliferation. ProTox-II analysis also indicates potential aryl hydrocarbon receptor binding. Notably, the PPIs omeprazole and lansoprazole may have a stronger association with cancer compared to pantoprazole and esomeprazole. Furthermore, we find that the H2 receptor blocker famotidine might be less associated with cancer compared to PPIs and could potentially serve as a safer alternative treatment for periods exceeding three months.

Future directions. Future directions could incorporate investigations into the mechanisms by which PPIs contribute to cancer development. While the reviewed evidence suggests a link between PPI use and certain types of cancer, the underlying biological mechanism remains unclear. Therefore, future research must focus on investigating the molecular pathways by which PPIs could promote cancer development and progression.

In the future, the long-term effects of PPIs could also be investigated using randomized control studies. Notably, the studies reviewed herein are observational and retrospective, rather than interventional, and do not establish any direct causality. Future research could focus on conducting randomized controlled trials with long-term follow-ups to better understand the particular risk of PPIs.

Given the risks associated with long-term PPI use, future studies could evaluate alternative treatments for acid reflux and related conditions, such as other drugs like H2R blockers, lifestyle changes, and diet adaptions.

In the future, PPIs could not be generalized into one pharmacological class that shares all side effects, and PPIs should be investigated separately for their risk of cancer. For example, omeprazole could contribute to cancer development much more than lansoprazole, or vice versa. Future research must distinguish between different PPI family members and establish individual safety profiles for each of the following drugs: omeprazole, lansoprazole, dexlansoprazole, pantoprazole, rabeprazole, and esomeprazole.

Future directions could also include the identification of high-risk patient subgroups. The risk of cancer associated with long-term PPI use may vary greatly and could depend on confounding factors like age, sex, lifestyle, obesity, genetics, and comorbid conditions. As such, future research could identify subgroups of high-risk patients and strive for personalized medicine strategies for managing acid reflux and related conditions. 

Finally, clear guidelines for PPI use could be established based on this evidence. For example, long-term PPI use is associated with the malabsorption of vital minerals (i.e., calcium, magnesium, and iron) and vitamins (i.e., B12) [62]. Thus, future guidelines should mandate specific dietary supplements accompanying long-term PPI treatment.

Limitations. Several studies and their reviews have proposed a link between long-term PPI use and an increased risk of cancer. Notably, these studies have several limitations and cannot definitively establish a cause-and-effect relationship between PPIs and cancer. In particular, short-term PPI use (<3 months) is not associated with an increased risk of cancer. 

Potential limitations to these studies include confounding factors, direct causality, and the absence of randomized controlled trials. For example, people requiring PPI treatment may have other risk factors for cancer, such as age, lifestyle, medical history, a high body mass index, eating disorders, stress, and smoking, potentially confounding the results. In addition, perhaps the increased risk of cancer is not caused by PPI use, but rather by the underlying medical pathology requiring PPI treatment. Also, there is a lack of randomized controlled trials which investigate the association of long-term PPI use and the risk of cancer, and to date, the cause-and-effect relationship has not been established. 

Nevertheless, the reviewed evidence suggests that long-term PPI use (>3 months) is more strongly associated with cancer, compared to short-term use (<3 months). The reviewed evidence also suggests a dose-dependent association, and that higher doses of PPIs are more strongly associated with the risk of cancer than lower doses. As such, physicians and healthcare professionals should consider and discuss the potential risks and benefits of long-term and high-dose PPI use with their patients, including relief from acid reflux and related conditions. 

Conclusions. Having looked at the epidemiologic evidence showing a causal relationship between PPIs’ long-term use and cancer risk, it is clear that there is an influence of both dosage and duration of use on the correlation. While the association remains clear, none of the studies have attempted to rank PPIs according to their relative risk. Here, we rank each PPI’s association with cancer risk based on PubMed citation counts, and find it to be lower for esomeprazole and pantoprazole and higher for omeprazole and lansoprazole. Notably, the PubMed citation count also ranks the cancer risk as higher with PPIs than with famotidine, an H2R blocker.

The mechanisms leading to this association remain unknown. Possible explanations involve changes in the gut microbiome, output of gastrin, the proliferation of enterochromaffin-like cells, and/or non-specific binding to protein targets associated with cancer. Additional studies need to be conducted for a plausible explanation of those mechanisms and the potential harm posed by individual PPI consumption in the long term.

Although PPIs have been effective in controlling gastrointestinal conditions, the very extended usage of the drugs may increase cancer risk. This review emphasizes the role of thoughtful clinical decision making as a vital aspect in finding the safest alternative for treating high stomach acid levels. The end objective is to give the most efficient and safe treatment for every patient with customized medical requirements.

## Figures and Tables

**Table 1:** Studies of long-term PPI use and cancer risk.

Population	Analysis	Comparison	Outcome	References
A total of 5589 patients diagnosed with colorectal cancer, receiving >30 pills of PPIs	Conditional logistic regression adjusted for multiple covariates	A total of 55,890 healthy controls, receiving >30 pills of PPIs	Long-term PPI use is not associated with increased risk of colorectal cancer	Sørensen and coworkers [32]
A total of 3,288,092 patients receiving PPIs for more than a year	Time-dependent Cox regression with all covariates	A total of 2,887,030 controls who had never used PPIs	Long-term PPI use is associated with a 25% increased risk of death	Al-Aly and coworkers [33]
A total of 3271 patients, after *H. pylori* infection, receiving PPIs weekly for at least one year	Cox proportional hazards model with propensity score adjustment	A total of 60,126 patients, after *H. pylori* infection, not receiving PPIs	Long term PPI use is associated with a 2-fold increased risk of gastric cancer	Leung and coworkers [13]
A total of 233,173 women diagnosed with cancer receiving PPIs for more than 60 days	Conditional logistic regression adjusted for potential confounders	A total of 932,692 healthy women not receiving PPIs for more than 60 days	Long-term PPI use is associated with a reduced risk of breast, ovarian, and cervical cancer	Yang and coworkers [16]
Data from 2396 records, including 5 retrospective cohort and 8 case-control studies	Meta-analysis of systematic search in Ovid MEDLINE, EMBASE, and Cochrane library	PPI use and gastric cancer development	Long-term PPI use for 1 and 3 years is associated with a 1.5 and 2.4-fold increase in the risk of gastric cancer, respectively	Misslewitz and coworkers [34]
Data from 12 observational studies	Meta-analysis of search in PUBMED, EMBASE, and Cochrane Central Register	PPI use and development of fundic gland polyps and gastric cancer	Long-term PPI use (>1 year) is associated with a 2.98 and 1.41-fold increased risk of fundic gland polyps and gastric cancer, respectively	Stehouwer and coworkers [14]
Data from 10 observational studies	Meta-analysis of search in PubMed, SCOPUS, Cochrane library, and Google scholar	PPI use and development of pancreatic cancer	Long-term PPI use is not significantly associated with the risk of pancreatic cancer	Jung and coworkers [35]

**Table 2:** Predicted association of proton pump inhibitors (PPIs) with cancer, neoplasm, and tumor.

Proton Pump Inhibitor (PPI)	Number of PubMed Citations		Normalized Association
	PPI	PPI and (Cancer OR Neoplasm OR Tumor)	
**Omeprazole**	13,796	1621	0.12 *
**Lansoprazole**	3232	390	0.12 *
**Pantoprazole**	2407	246	0.10 *
**Esomeprazole**	2044	214	0.10 *
**Rabeprazole**	1543	143	0.09
**Dexlansoprazole**	166	13	0.08
**Famotidine (H2R blocker)**	2519	210	0.08 *

* Statistically significant.
